# Germline *VWF*/*MPRIP* and somatoplasm *FGA* variants synergically confer susceptibility to non-traumatic osteonecrosis of the femoral head

**DOI:** 10.1038/s41598-023-30260-4

**Published:** 2023-02-22

**Authors:** Dawei Wang, Longchao Gu, Juan Zheng, Qiang Zhang, Qi Xu, Rongrong Li, Da Song, Chengzhi Ha, Qianqian Zhang, Han Yin, Mingtao Xu, Hongmin Wang, Wei Li, Zhengfeng Yuan, Cuncun Yang, Mingliang Gu

**Affiliations:** 1 Department of Orthopedic Surgery, Liaocheng People’s Hospital, Liaocheng, 252000 Shandong China; 2 Joint Laboratory for Translational Medicine Research, Liaocheng People’s Hospital, Liaocheng, 252000 Shandong China

**Keywords:** Genomics, Mutation, Sequencing

## Abstract

Non-traumatic osteonecrosis of the femoral head (ONFH) relies on multiple pathogenic factors, including intravascular coagulation, osteoporosis and lipid metabolism disorders. Despite extensively explored from various aspects, genetic mechanism underlying non-traumatic ONFH has not been fully elucidated. We randomly collected blood and necrotic tissue samples from 32 patients with non-traumatic ONFH as well as blood samples from 30 healthy individuals for whole exome sequencing (WES). Germline mutation and somatic mutation were analyzed to identify new potential pathogenic genes responsible for non-traumatic ONFH. Three genes might correlate with non-traumatic ONFH: VWF, MPRIP (germline mutations) and FGA (somatic mutations). Germline or somatic mutations in VWF, MPRIP and FGA correlate with intravascular coagulation, thrombosis, and consequently, ischemic necrosis of the femoral head.

## Introduction

ONFH, also known as avascular necrosis of the femoral head, is a pathologic condition in which damaged or interrupted blood supply leads to osteocyte apoptosis, structural changes, abnormal weight-bearing of the hip joint, and subsequent collapse and necrosis. This group of disorder is mainly divided into traumatic and non-traumatic ONFH^1,2^. Non-traumatic ONFH is more common in men aged 20–50 years. Due to insidious occurrence, its diagnosis has been always delayed, making it extremely difficult to be treated^3,4^. If a necrosis area is ≥ 30% and untreated, non-traumatic ONFH would progress to end-stage arthritis, resulting in severe damage to the hip joint and thus impaired, even disabled functions. Non-traumatic ONFH can be attributed to multiple pathogenic factors, including intravascular coagulation, osteoporosis and lipid metabolism disorders^5–7^

*COL2A1* was the first susceptible gene reported to be associated with ONFH, possibly related to chondrodysplasia and growth arrest, or even spinal deformity and femoral head dysplasia^8,9^. *SOX9* rs1042667 and rs12601701 were closely related to an increased risk of ONFH^10^. Theoretically, *SOX9* might physically interact with *RUNX2* and thereby inhibit osteogenesis of RUNT-domain transcription factor^11,12^. In addition, *MMP8* rs11225394, *MMP3* rs650108 and rs522616, as well as *MMP14* rs2236302 significantly correlated with non-traumatic ONFH^13–15^. Furthermore, *clotting factor V*, *MTHFR* and *PAI-1* variants correlated with high coagulation or low fibrinolysis, which might promote intravascular coagulation or weaken thrombolysis, causing ischemic necrosis of the femoral head^16–18^. Additionally, immune factor *CR2* variant correlated with non-traumatic ONFH^19^. *IL-1*^20^ polymorphism and *TNF-α*^21^ genotypes might increase the risk of femoral head necrosis. Although non-traumatic ONFH has been extensively explored from different aspects, its genetic mechanism has not been fully elucidated.

To fill in the gap in knowledge, we collected tissue and blood samples from 32 patients with non-traumatic ONFH as well as blood samples from 30 healthy individuals for whole exome sequencing. We explored new potential pathogenic genes for non-traumatic ONFH based on germline and somatic mutations. Through gene function annotation and signaling pathway analysis, potential roles of embryonic genetic (germline) and somatic mutations in non-traumatic ONFH pathogenesis were explored, providing molecular targets for clinical diagnosis and treatment.

## Results

### Germline mutation gene identification

Sequencing data of blood samples from 32 patients were analyzed. The average reads were 85,074,024.25, and the average depth was 53.5 (Supplementary Table S4). 30 healthy people's blood samples, the average reads were 106,522,490.2, and the average depth was 70.4 (Supplementary Table S4). Genes and sites with the minor allele frequency of > 0.01 were analyzed by Plink correlation. Subsequently, 12 genes related to femoral head necrosis were discovered (*p* < 0.01) (Supplementary Table S1). Frequency statistics were conducted for genes and sites with the minor allele frequency of ≤ 0.01. 19 genes related to femoral head necrosis were discovered (with a frequency > 15%) (Supplementary Table S2). 19 genes met the following criteria: (1) at least 2 of the 4 software (SIFT, Polyphen2_HDIV, Polyphen2_HVAR, LRT) predicted harmful SNVs and Indels; (2) the variant frequency could be identified in all samples for > 15%. Totally, 31 germline mutant genes were screened.

### Somatic mutation gene identification

**Figure 1:**
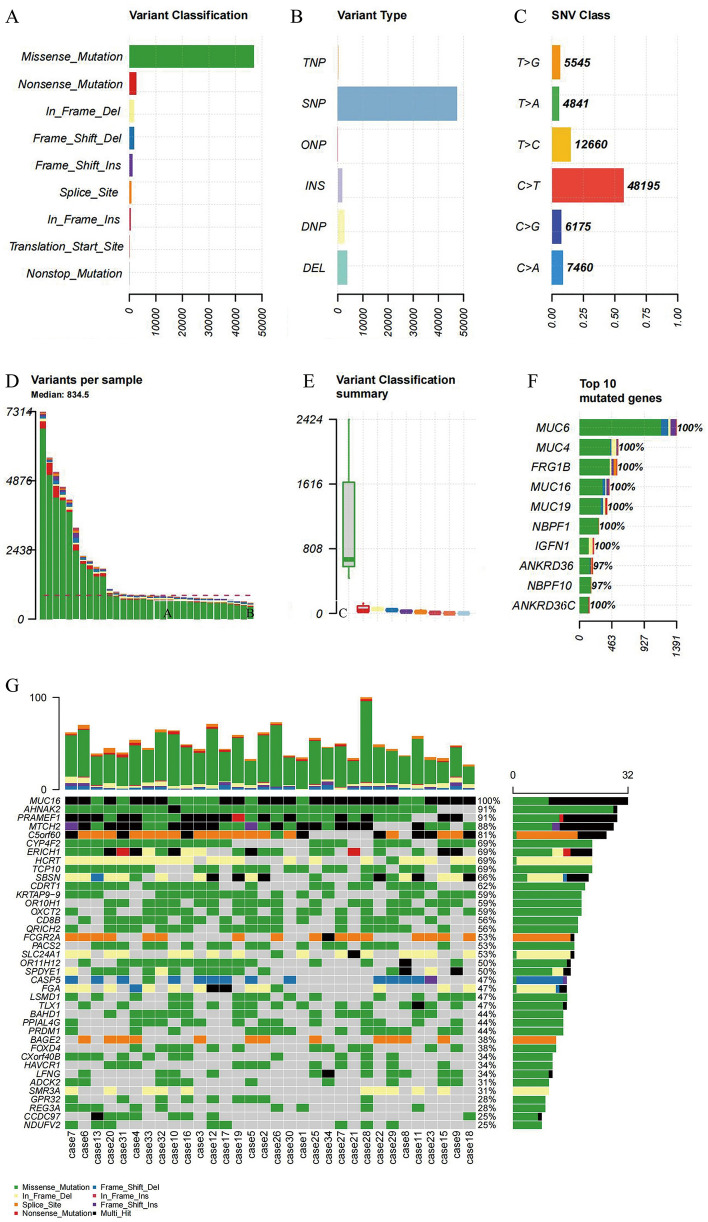
Somatic mutation analysis. (**A**) Variant classification, x represents quantity, y represents classification (**B**) Variant types, x represents quantity, y represents type (**C**) SNV classification, x represents ratio, y represents class (**D**) number of variants in each sample (**E**) Box plot of variant types, x represents quantity, y represents classification (**F**) quantity and frequency of the top10 mutated genes (**G**) Map of high-frequency variant genes. top: quantity of variant types in each sample, x represents sample, y represents the number of variants; bottom: types of genetic variants in different samples, x represents a sample, y represents a gene’s name; right: frequency of genetic variants in different samples. x represents frequency of each gene in a sample, and y represents gene’s name.

Sequencing data of necrotic tissues samples from 32 patients with non-traumatic ONFH were analyzed. The average reads were 92,402,683.94, and the average depth was 54.5 (Supplementary Table S4). Blood samples from 32 patients with non-traumatic ONFH, the average reads were 85,074,024.25, and the average depth was 53.5 (Supplementary Table S4). Through analyzing sequencing data, 94,062 somatic mutations were detected, comprised 47,025 missense, 29,636 silent, 814 splice-site, 2519 nonsense, 7973 RNA, 5083 indel, 27 nonstop and 58 transcription start points (Fig. 1A). 118 focal CNVs were obtained including 48 amplifications and 70 deletions (q < 0.25) (Supplementary Fig. S1). There were 49,629 SNPs, 1578 insertions and 3505 deletions (Fig. 1B). The main point variants were C > T, T > C and C > A, with a rate of 56.7%, 14.9% and 8.7%, respectively (Fig. 1C). The highest number of variants in a single sample was 7,314, with an average of 834.5 (Fig. 1D). The most significant type of variants was missense mutation (Fig. 1E). The frequency of variants in the top 10 genes was over 97% in the sample (Fig. 1F). Subsequently, using MutSigCV, 16,657 mutated genes were calculated (Supplementary Table S3), and 39 high-frequency variant genes (q < 0.01) were identified (Fig. 1G).

### Candidate gene identification

**Figure 2:**
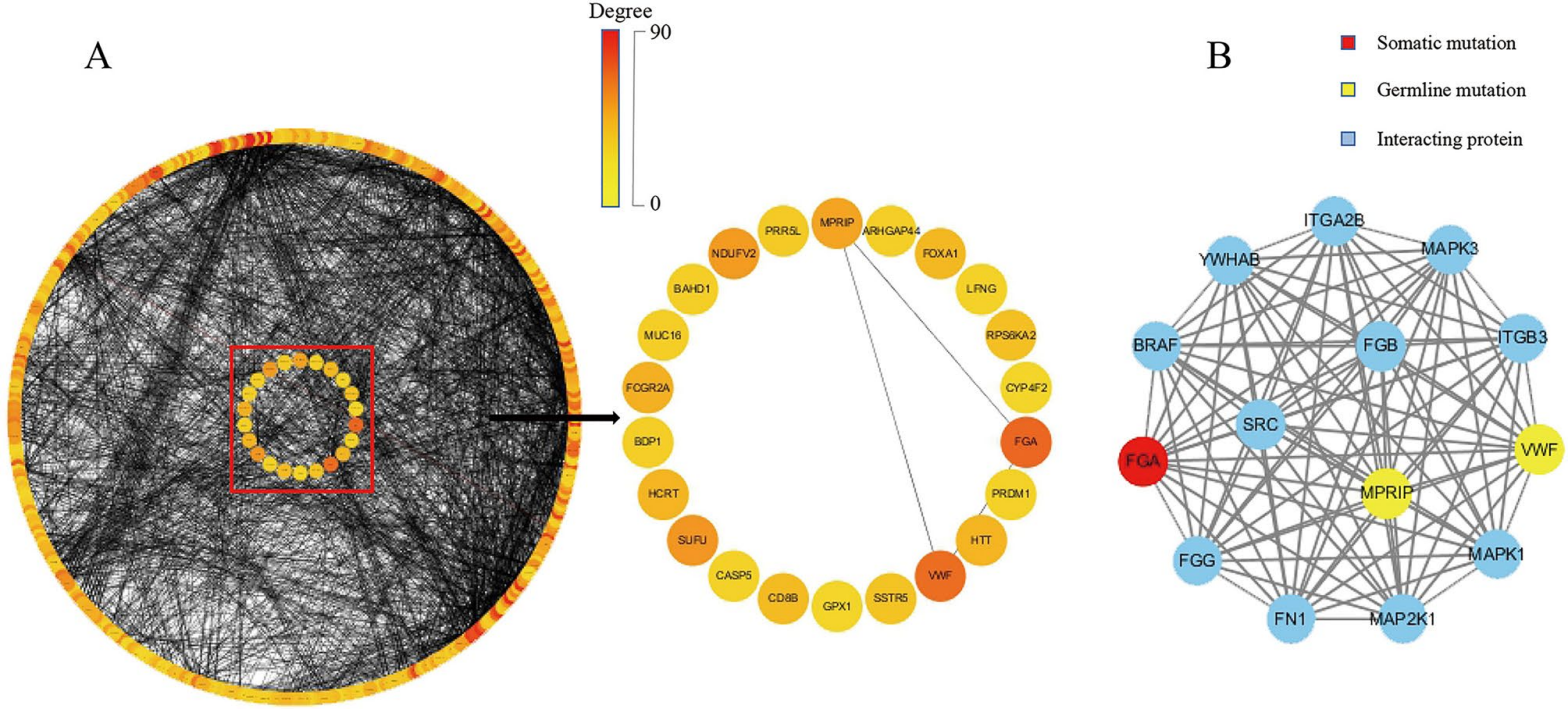
PPI network and functional modules. (**A**) PPI network. left: 436 nodes and 2888 edges. The outer circle represents interaction proteins whereas the inner circle represents candidate genes. right: Among 22 candidate genes, *FGA* and *VWF* have the highest Degree, and interact with MPRIP. The color indicates a change in Degree. The darker color indicates greater Degree and more important function node in the network (**B**) *VWF*, *MPRIP* and *FGA* belong to the same function module. yellow, red and blue represents germline mutation, somatic mutation, and interaction protein, respectively.

Based on HTRIdb and HPRD databases, potential interaction proteins encoded by 31 germline mutant genes and 39 somatic mutant genes were queried. Experimentally verified high quality proteins that interacted with candidate genes were selected. In HPRD, 220 pairs of protein interactions were obtained (Supplementary Table S6). In HTRIdb, 615 pairs of protein interactions were obtained (Supplementary Table S7). Protein–protein interaction (PPI) network analysis (interaction reliability > 0.4) identified 11 germline mutant genes and 11 somatic mutant genes. *FGA* (Degree: 31) and *VWF* (Degree: 29) carried the highest degrees (Fig. 2A). Using MCODE, *FGA*, *VWF* and *MPRIP* were classified into the same functional module (Credibility score:14) (Fig. 2B). *VWF* had nonsynonymous SNV mutations in the same location in 30 patients, including heterozygous mutations in 6 patients whereas homozygous mutations in others. *MPRIP* had a non-frameshift deletion mutation in the same location in 10 patients: 9 patients with biallelic mutations and 1 patient with homozygous mutation. *FGA* exhibited an in-frameshift deletion mutation in the same location in 12 patients, all of whom were heterozygous (Supplementary Table S5).

### Candidate gene function analysis

**Figure 3:**
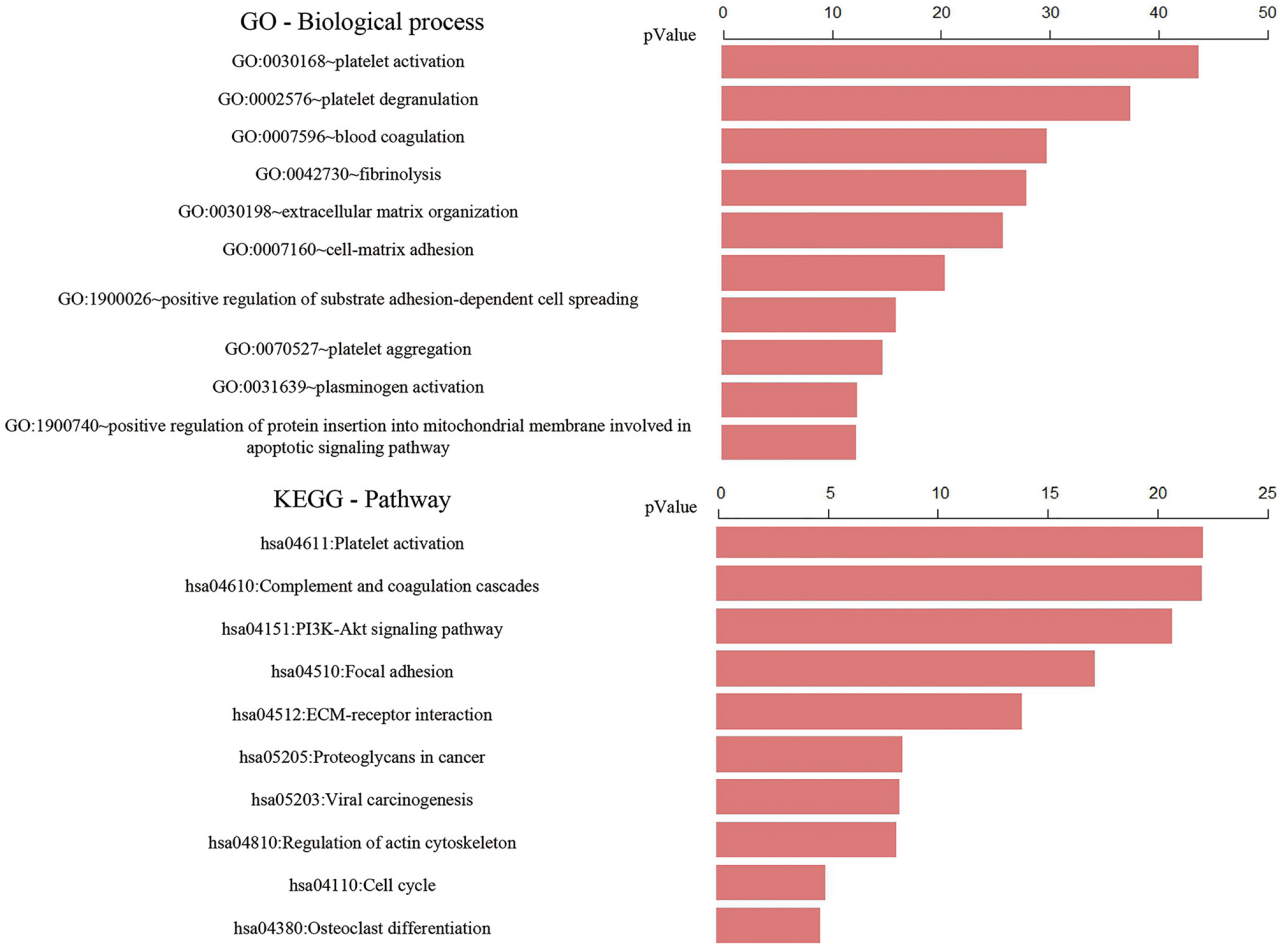
GO biological process and KEGG signaling pathway. top: GO biological process; bottom: KEGG signaling pathway.

Based on biological processes and signal pathways (Fig. 3), Von Willebrand factor (VWF) participates in degranulation and activation of platelets, promotes adhesion of platelets to injured sites, and enables blood coagulation to achieve hemostasis. Fibrinogen Alpha Chain (FGA) contributes to fibrin formation and exerts hemostatic effects. *VWF* variant (c.A1451G: p.H484R) is located in exon 13, and within the VWFD2 domain. VWFD domain is required for blood clotting factor VIII binding, and for normal multimerization of VWF. *FGA* variant (c.904_1020del: p. Pro302_Gly340del) is not located in its domain. We speculated that *VWF* variants might cause intravascular coagulation and thrombosis, insufficient blood supply to the femoral head, and subsequent avascular necrosis of the bone. Simultaneously, *FGA* variants might affect fibrin decomposition and liquefaction, so that intravascular thrombus couldn’t be dissolved. If the thrombus cannot be cleared in time, it might result in vascular occlusion, which aggravates osteocyte avascular necrosis. Myosin Phosphatase Rho Interacting Protein (MPRIP) regulates transformation through acting on cytoskeleton. *MPRIP* variants exist in different transcripts, all of which are not located in its domain. *MPRIP* variants might correlate with abnormal cytoskeleton and distorted morphology of bone cells. Intravascular coagulation and thrombosis might aggravate high-pressure in the bone marrow cavity, which triggers structural changes of the femoral head.

Those genes and interacting proteins were annotated and enriched in pathways, such as blood coagulation, platelet activation, platelet degranulation, and focal adhesion (Fig. 3). Notably, *FGA*, *VWF* and *MPRIP* variants correlated with intravascular coagulation and thrombosis, as well as impaired mechanosensory functions of osteocytes. Thus, both coagulation and thrombus participate in avascular necrosis of osteocytes. Coagulation and thrombus might aggravate compression of the bone marrow cavity, leading to structural abnormality of the femoral head.

## Discussion

We have performed WES on necrotic tissues and blood samples from 32 patients with non-traumatic ONFH as well as blood samples from 30 healthy controls. To explore pathogenesis of non-traumatic ONFH, we have examined perspective interactions between germline mutations and somatic mutations. Through in-depth mining and systematic analysis, 3 candidate genes associated with non-traumatic ONFH have been identified: *VWF*, *MPRIP* (germline mutations) and FGA (somatic mutations).

VWF is the partner of coagulation factor VIII, which contributes to platelet’s degranulation, activation, migration and adhesion in response to injury, thus achieving hemostasis. FGA is cleaved by proteases, to produce a monomer or to polymerize with fibrinogen β (FGB) and fibrinogen γ (FGG) and to form insoluble fibrin matrix. FGA is one of the main components of blood clots, which plays an important role in hemostasis. *FGA* variant was proposed to generate defective fibrin fibers, related to family history of recurrent thrombosis^22^. Intravascular coagulation is a direct cause of ONFH. The most common pathophysiological abnormalities of non-traumatic ONFH include intravascular coagulation and microcirculation thrombosis^23^. Hypercoagulation and low fibrinolysis are common in patients with ONFH, which jointly contribute to thrombosis occlusion of blood vessels in the femoral head, and thus aggravating avascular necrosis of osteocytes^24^. *VWF* and *FGA* variants may promote platelet’s degranulation and activation. Subsequently, fibrin could not be liquefied and decomposed, so intravascular coagulation and thrombosis of the femoral head would be expected. Vascular occlusion results in avascular necrosis of osteocytes and thus osteopenia, eventually causes non-traumatic ONFH. *MPRIP* encodes myosin phosphatase RHO-interacting protein, which is involved in cytoskeleton regulation and extracellular matrix adhesion. MPRIP plays multiple roles in cellular migration, adhesion, division and differentiation^25,26^. *MPRIP* variants may correlate with cytoskeleton and morphology, loss of or reduction in cell sensitivity, sparse trabecular bone and collapse of the femoral head. The superposition and persistence of interactions among *VWF, FGA* and *MPRIP* are expected to mediate avascular necrosis of the femoral head.

Based on GO Term and KEGG pathway analysis, *FGA, VWF* and *MPRIP* are involved in coagulation, platelet degranulation and adhesion plaques. We speculated that superposition of germline mutated *VWF* onto somatic mutated *FGA* might mediate coagulation and thrombosis of the femoral head vessels, consequently, avascular necrosis of osteocytes. Simultaneously, *MPRIP* variants might distort cytoskeleton and morphology, leading to osteopenia, eventually collapse and ischemic necrosis of the femoral head. Therefore, we propose that non-traumatic ONFH is attributed to interactions between germline mutations and somatic mutations.

Several limitations should be kept in mind. This study was focused on non-traumatic necrosis of the femoral head, excluding alcohol-related and hormone-induced necrosis of the femoral head. The number of cases is low, and most cases were sporadic. Due to a small sample size, readouts might be biased. A large number of samples need to be collected to ensure the credibility of the results. Moreover, candidate pathogenic genes should be verified with functional experiments. Nevertheless, this study provides references for a better understanding of nontraumatic ONFH. In addition, identification of candidate pathogenic genes will provide promising molecular targets for clinical diagnosis and treatment of ONFH.

## Methods

### Clinical research

Patients with non-traumatic ONFH, who were hospitalized for surgical treatment in Liaocheng People’s Hospital, were randomly selected according to the guidelines for clinical diagnosis and treatment of adult femoral head necrosis (2019)^27^. All patients had no history of alcoholism and chronic use of hormones. Hormonal osteonecrosis of the femoral head and alcoholic osteonecrosis of the femoral head were excluded. Patients were diagnosed independently by 2 clinicians based on X-ray, CT or MRI imaging systems. The degree of femoral head necrosis in patients was stage IV. The necrotic frozen tissues or formalin-fixed and paraffin-embedded (FFPE) sections were derived from hip replacement surgery. After hematoxylin–eosin staining, pathological diagnosis was performed under light microscope. The remaining tissues were frozen in liquid nitrogen within 30 min post-operation. Simultaneously, 2 ml of peripheral blood was collected into an EDTA anticoagulation tube and stored at −80 °C. In addition, 30 healthy people matched with those patients in terms of age and gender were selected as controls. 2 ml of peripheral blood from a healthy control was collected and stored in a refrigerator at −80 °C. According to the Declaration of Helsinki, this study was approved by the Ethics Committee of Liaocheng People's Hospital. Each participant had signed informed consent form prior to sample collection.

**Figure 4:**
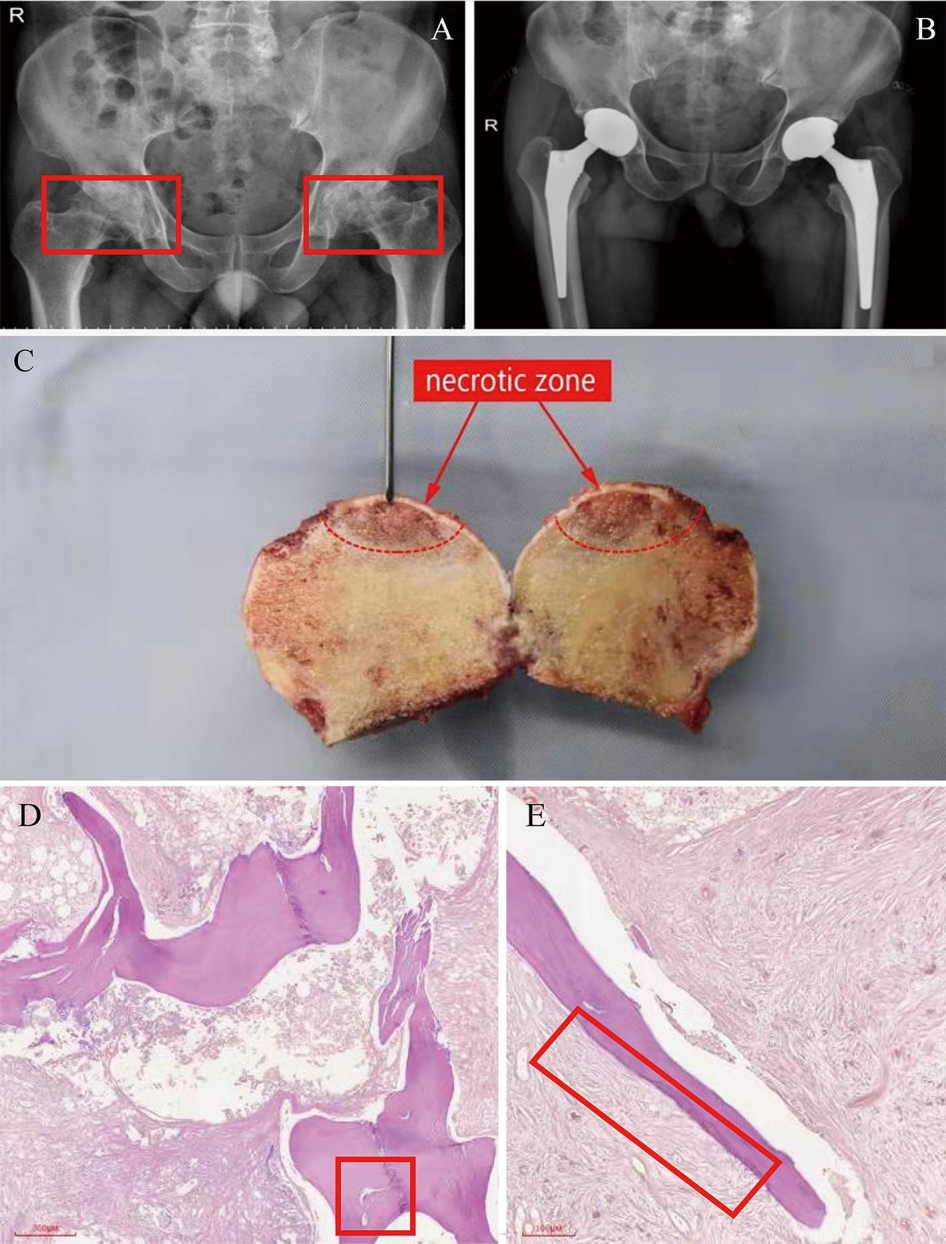
Imaging system for clinical diagnosis. (**A**) Preoperative pelvic frontal radiograph (X-ray). Bilateral femoral heads were flat, with multiple cystic low-density shadows and narrowed joint space (red boxes on both sides) (**B**) Implanted prosthesis on pelvis orthographic film (X-ray) (**C**) Diseased tissue (with a red arrow pointing to necrosis). (**D**) Morphological abnormality in bone trabecular (left red frame) (40 ×). (**E**) Bone trabecular necrosis (right red box) (100 ×).

32 patients with non-traumatic ONFH included 26 males and 6 females, aged at 59.56 ± 9.517 years. 4 (12.5%), 8 (25%) and 20 patients (62.5%), respectively, suffered from the left, the right, and bilateral femoral head necrosis. The diseased tissue was localized through radiographic, pathological (Fig. 4 A-C) and cytological images (Fig. 4 D-E).

### Whole exome sequencing (WES)

Genomic DNA was extracted from whole blood by using QIAamp DNA blood Mini Kit (250) (Qiagen, Germany). Genomic DNA was extracted from tissue sample by using QIAamp DNA Mini Kit (250) (Qiagen, Germany). Tissuelyser II (Qiagen, Germany) was used to grind the femoral head tissue using 0.5 mm steel ball, at 30 frequency/s, 5 min/time, for 2 times.Quality control was performed by using a nanodrop (Thermo Fisher Scientific, USA), with an OD 260/280 ratio of 1.8–2.0^28^.

DNA libraries of blood and tissue were constructed in the same way. Firstly, DNA was interrupted into ~ 200 bp fragments with Covaris S220. Breaking parameters were set up as follows: Duty factor 10%; Peak Incident Power 175; Cycles per Burst 200; Treatment time 360 s; and Bath Temperature 4–8 °C. Agilent 2100 quality control was performed on fragmented DNA.

Secondly, Agilent Sureselect DNA Targeting Sequence Capture Kit (Agilent SureSelectXT Human All Exon V6) was applied for library preparation as follows: (1) End repair was performed on fragmented DNA, where A was added to 3' end, and all gaps were connected with adapters. After each step, AMPure XP beads were used for purification. (2) Polymerase chain reaction (PCR) was performed with an amplification volume of 50 µl. The program was set up as follows: 98 °C pre-denaturation for 2 min; 98 °C denaturation for 30 s, 65 °C annealing for 30 s, 72 °C extension for 1 min, totally 10 cycles; 72 °C extension 10 min; and 4 °C, hold. The product was purified with AMPure XP beads. (3) Amplified DNA was hybridized and placed at 65 °C for 16–24 h. (4) After hybridization, stranded penicillin magnetic beads were applied for probe capture and PCR amplification, with an amplification volume of 50 µl. The program was set up as follows: pre-denaturation at 98 °C for 2 min; denaturation at 98 °C for 30 s, annealing at 57 °C for 30 s, and extension at 72 °C for 1 min; totally 12 cycles; 72 °C extension for 10 min; and 4 °C, hold. (5) AMPure XP beads were used for purification, whereas Aglinet 2100 for quality control. The fragment size was 250–350 bp, and thus, library preparation was completed.

Finally, Nextseq 500 (Illumina) was applied for PE75 sequencing.

### Data quality control and general analysis

Trimmomatic (V 0.36) was used to remove the index and to filter out low-quality bases. BWA software (V 0.7.17) was employed to map the filtered reads with the hg19 human genome as a reference. Picard was applied to mark and delete repeated sequences. GATK4.1.8.1 Recalibration was integrated for local mapping whereas base recalibration for the variants calling program.

### Germline mutation analysis

Haplotype Caller was performed to call variants of patients’ and healthy controls’ blood samples. Variant Recalibrator and Apply Recalibration were applied for quality control and filtering (Broad Institute)^29^. To screen the genes and sites with a minor allele frequency of > 0.01 in patients’ blood samples, Plink association analysis was used to determine significant genes and sites (*p* < 0.01). 1000 genome data were used as a reference in the calculation and filtering process using Plink. The frequency of individual genes and sites with a minor allele frequency of ≤ 0.01 in the sample was calculated (with a frequency > 15%). ANNOVAR was conducted to annotate each variant to predict potential functional impact^30^.

### Somatic mutation analysis

MuTect2 (Broad Institute)^29^ was performed for somatic mutation calling: firstly, the paired peripheral blood sample data were processed to construct a PoN and to provide filtering support for calling somatic mutations;Secondly, preliminary somatic mutation calling was performed to correct and filter those original mutations. The resulting somatic mutations were annotated by VEP. Variance analysis was performed to predict potential functional impact of each variation^31^. Vcf2Maf software was used to convert annotated VCF file into MAF file, whereas maftools software package was applied to visualize variation distribution. MutSigCV^32^ (version 1.41) was used to identify somatic SNV and InDel variant and to predict those genes with high-frequency variants (q < 0.01). 1000 genome data were used as a reference in the calculation and filtering process using call variants.

### Copy number variation (CNV) analysis

Using CNVKit^33^, Binary Alignment Map of 32 matched necrotic tissues and blood samples with the hg19 human genome as a reference was established. CNV fragments of 32 samples were obtained and calculated via GISTIC (q < 0.25)^34^.

### Construction and analysis of PPI network

HTRIdb and HPRD databases were employed to query interaction proteins among those identified genes. High quality proteins that were verified to interact with candidate genes were selected. A union of the results from two databases was taken. STRING^35^ was performed to construct a PPI network (score > 0.4). Cytoscape v3.7.2^36^ was applied for network visualization. MCODE^37^ was conducted to detect closely connected network submodules in a PPI network.

### GO and KEGG enrichment analysis

Database for Annotation, Visualization and Integrated Discovery (DAVID) (version 6.8)^38^ was used to annotate the functions of proteins and genes which could be extracted and analyzed. GO (Gene Ontology) functional enrichment analysis and KEGG^39^ (Kyoto Encyclopedia of Genes and Genomes) pathway annotations were performed on candidate genes, with a *p* < 0.05 as the minimum standard for any significant difference.

## Supplementary Information

Supplementary Information 1.

Supplementary Information 2.

Supplementary Information 3.

Supplementary Information 4.

Supplementary Information 5.

Supplementary Information 6.

Supplementary Information 7.

Supplementary Information 8.

## Data Availability

Primary data which are held in a secure Research Environment, are available to registered users. Please search the database https://db.cngb.org/search/project/CNP0002252/ for detailed information. All other relevant data from this study will be available from the corresponding authors upon reasonable request.
