# Supplementary figures and images for: Germline VWF/MPRIP and somatoplasm FGA variants synergically confer susceptibility to non-traumatic osteonecrosis of the femoral head
Source: Sci Rep. 2023 Feb 22;13:3112. doi: 10.1038/s41598-023-30260-4 (PMC9946931; doi:10.1038/s41598-023-30260-4)

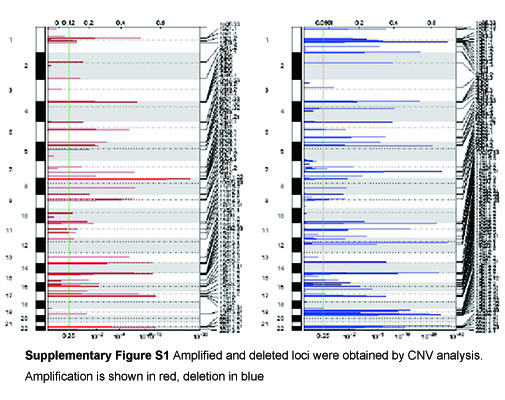

Supplement: Supplementary file 1 — Supplementary Information 1. [file 41598_2023_30260_MOESM1_ESM.jpg]
